# Higher-order moments spillovers among energy, carbon and tourism markets: Time- and frequency-domain evidence

**DOI:** 10.1371/journal.pone.0313002

**Published:** 2024-11-14

**Authors:** Wang Gao, Shixiong Yang

**Affiliations:** 1 School of Finance, Hebei University of Economics and Business, Shijiazhuang, China; 2 School of Statistics, Renmin University of China, Beijing, China; Kinnaird College for Women, PAKISTAN

## Abstract

This paper uses the GJRSK model to estimate the high-order moments of energy (oil, natural gas, and coal), the carbon market, and tourism stocks. Then, it utilizes a novel TVP-VAR time-frequency connectedness approach to examine higher-order moments spillovers among them. The results show a strong connectedness among the three markets. The energy market is the emitter of volatility, skewness and kurtosis spillovers; tourism stock is the receiver; and the carbon market is the transmitter. From a time-domain perspective, the higher-order moments spillovers of the three markets are time-varying, especially during extreme periods, where the energy market’s spillover effects on tourism stocks are significantly enhanced, indicating that tourism stocks bear a greater risk at leptokurtosis and fat-tail moment. From a frequency-domain perspective, the long-term asymmetric spillovers of oil, natural gas, and tourism markets on the carbon market are more pronounced than the short-term. Moreover, the COVID-19 pandemic exacerbated the higher-moment spillovers of energy and tourism stocks on the carbon market. Meanwhile, the Russia-Ukraine conflict led to extreme risk transmission within the energy market. These findings have significant implications for cross-industry investors and green finance risk management.

## 1. Introduction

In the current context of urgent global warming and climate change, the connection between energy and carbon markets has become a major topic of discussion in the academic community [1,2]. Literature suggests that the information spillover and linkage effects between the two markets are continuously strengthening [3,4] and gradually diffusing into the green and low-carbon industries, including power, renewable energy, and clean technology [5,6]. The transformation towards green and low-carbon practices is essential for various industries to reach carbon peak and neutrality. Tourism, which has always been regarded as a sunrise industry with low pollution emissions and high returns, plays a vital role in the transition. However, the industry’s energy consumption and greenhouse gas emissions cannot be ignored. On the one hand, the growth of tourism heavily relies on energy, particularly in transportation and accommodation, which require significant electricity and fuel consumption [7]. On the other hand, the sustainable development of the tourism industry requires an environmentally friendly approach, creating a strong demand for low-carbon transformation [8]. In this context, it is vital for investors and policymakers to study the close relationship among energy, carbon, and tourism markets in order to understand potential systemic risks.

In recent years, scholars have carried out continuous exploration of the relationship among energy, carbon and tourism markets and found various links such as cointegration, causality, and spillover effect of its return/volatility [9–11]. It suggests that the relationship between the three markets is becoming increasingly interconnected. However, Khashanah, Simaan [12] argued that the two-dimensional framework limited to ‘mean-variance’ will bring new challenges to investors and policymakers concerned about systemic risks. Further, some scholars have found that energy and carbon assets have volatility clustering, asymmetry, and fat tail characteristics [13–15]. They indicated that ignoring the time-varying characteristics that may exist at high-order moments, such as skewness (third-order moment) and kurtosis (fourth-order moment), is not conducive to asset pricing and portfolio. Regrettably, no academic has explored the high-order moments relationship between energy, carbon and tourism markets, which is not conducive to portfolio allocation and risk management.

Therefore, this paper aims to examine the spillovers of higher-order moments in these three markets. On the one hand, existing studies have found the skewness spillover effect between energy and carbon markets [13,14], but whether the tourism market will have skewness spillover effects with the two markets remains unstudied. Most research on the tourism market assumes that the conditional distribution of asset returns is normally distributed [16,17]. Considering the leverage and volatility feedback effects [18] and expected volatility changes, tourism stocks may have inconsistent responses to the rise and fall of energy and carbon prices, which requires further exploration. On the other hand, frequent global extreme climate and international geopolitical conflicts have become ‘black swan’ events that caused a sharp jump and mutation in the energy, carbon, and tourism markets in a short period [19–21]. Excess kurtosis can rapidly spread risk across markets due to the close relationship between energy, carbon, and tourism markets. Therefore, exploring the high-order moment spillover effects among the three markets is necessary.

To figure out underlying relations, this paper first employs the Glosten, Jagannathan, and Runkle GARCH (GJR) model to estimate the high-order moments of energy, carbon and tourism markets [22]. This method describes the conditional volatility (skewness and kurtosis) in a straightforward form by extending the GARCH model, which is important in risk-based portfolios with higher moments. Then, this paper investigates higher-order moments spillovers among the three markets by employing a novel TVP-VAR time-frequency connectedness approach proposed by Chatziantoniou, Gabauer [23], which builds on the work of Baruník and Křehlík [24] and Antonakakis, Chatziantoniou [25]. The TVP-VAR time-frequency connectedness approach makes it possible to decompose connectedness into short- and long-run components. This approach allows us to analyze how the markets are connected over different periods and how skewness and kurtosis risk are transferred in the three-market system. It also helps us understand the dynamic spillover connectedness among the markets during recent crises such as the ’COVID-19 pandemic’ and the ’Russia-Ukraine war’, which may directly affect the information spillover structure of energy, carbon, and tourism markets.

This study contributes to the existing literature in two ways. Firstly, to the best of our knowledge, this is the first study to comprehensively investigate the time-frequency connectedness among energy, carbon, and tourism markets from the higher-order moment perspective. Specifically, the skewness connectedness can reveal how asymmetric risk spreads across three markets, while the kurtosis results reveal whether and to what extent extreme events in one market spill over into another. In the global era of frequent geopolitical conflicts and major crisis events, the higher-order moment results shed new light on analyzing the spillovers among three markets and highlight the significance of the higher-order moments in the decision-making procedure of diversified investment and risk management. Secondly, this research attempts to uncover the tri-variate systemic nexus mechanism by using a novel TVP-VAR time-frequency connectedness method of Chatziantoniou, Gabauer [23], providing a new research perspective for the study of cross-market spillovers. Exploring dynamic transmission mechanisms across time scales is essential to market participants with various investment horizon preferences.

The study uncovers significant discoveries regarding the high-order moment spillovers among energy, carbon, and tourism markets. It reveals that the energy market serves as a net exporter of volatility, skewness and kurtosis spillovers, while tourism stocks are net recipients. Notably, the connectedness among these markets is time-varying and intensifies during crises, such as the COVID-19 pandemic and the Russo-Ukrainian War. Furthermore, while short-term spillovers are more pronounced for returns and volatility, high-order moment spillovers—specifically skewness and kurtosis—exhibit greater long-term impacts, highlighting sustained risks in tourism stocks linked to energy and carbon. These findings have significant implications for emerging asset portfolios, emphasizing the need for careful consideration of interconnected risks.

The rest of the paper is arranged as follows. Section 2 presents a literature review. Section 3 describes the study’s data and methodology and offers some preliminary statistics. Section 4 presents and discusses the study’s empirical findings. Finally, Section 5 presents concluding remarks and policy implications.

## 2. Literature review

### 2.1 Energy and carbon markets

A significant body of literature has demonstrated the close relationship between the carbon and energy markets [26–28]. When energy prices decline, the demand will increase, increasing carbon emissions and prices. Either the income effect or the substitution effect can explain this. Zhang and Sun [29] explored the dynamic volatility spillover effect between energy and carbon markets. They found that the time-varying spillover is transmitted from the coal to the carbon market and then to the natural gas market. Gong, Shi [3] also showed that the spillover effect between carbon and fossil energy markets is time-varying, time-lag, and cyclical, usually lasting for three weeks. Su and He [28] explored the quantile relationships between carbon futures and energy markets and constructed an investment portfolio. In addition, many studies have reported asymmetric spillover effects in energy and carbon market returns and volatility [30–32]. Some studies have also examined the spillover effects of energy and carbon markets on other markets [33,34], indicating the risk characteristics inherent to these markets.

However, since both energy and carbon assets have significant financial attributes [35], classical analysis based on return/volatility is not conducive to determining whether extreme prices for energy and carbon assets will affect each other, namely asymmetry and fat tail risk [36]. Researchers believe this higher-order moment information is the prominent risk investors face and the primary source of systemic risk that policymakers need to consider [37,38]. Some empirical studies showed that the interaction between energy and carbon markets is heterogeneous on different time scales. For example, Dai, Xiao [36] revealed the multiscale interplay of higher-order moments between the carbon and energy markets. They indicated that the interplay of higher-order moment enhances a bullish market, and carbon is a good hedge against higher-order temporal risk in short time scales. Studies have indicated that the heterogeneity of higher-order moments spillovers in energy and carbon markets at different time scales [13,14], and people are increasingly interested in this non-Gaussian behavior [39]. However, no research has revealed the connection between energy and carbon markets from a high-order moment perspective, which is detrimental to investors’ asset allocation and risk management. Therefore, it is necessary to study the interaction of third-order and fourth-order moment characteristics between two markets.

### 2.2 Energy and tourism markets

Some transmission channels are proposed in the theoretical framework to explain how energy price affects tourism stock. The first channel is the stock valuation channel [40,41], which treats energy as a factor of production. Fluctuations in energy prices have changed tourism enterprises’ marginal production costs and profit margins. Traditional stock valuation methods show that current stock prices represent the future discounted cash flow. Therefore, changes in energy prices will affect the expected cash flow. For example, tourism stock prices will fall when oil prices rise due to lower expected cash flows [42,43]. The second channel is inflation caused by energy prices, which can increase domestic prices and tighten market liquidity. Since tourism and leisure markets are elastic, they are more vulnerable to economic contraction and reduced demand, thus reducing stock returns [44,45].

Empirical research has widely demonstrated the close relationship between tourism and energy. For example, Nižić, Grdić [46] found a causal relationship between tourism and energy consumption in countries with similar economic climates and determined that increasing the number of tourists would increase energy consumption. By expanding the sample group, Bhuiyan, Zaman [47] suggested that international tourism increased the exhaustion of energy resources and carbon emissions of various countries. Tourism also has a long-term impact on energy consumption. Feng, Sun [48] said that oil prices significantly impact China’s tourism market, and its net effect is mainly concentrated in the medium and long term, which can explain the significant changes in China’s tourism stock. Some scholars have begun to focus on the role of renewable energy in driving tourism economic growth. They highlight how investments in renewable energy sources can enhance sustainability in tourism [49,50]. According to current research, the relationship between oil and tourism has been well studied, while other energy, such as natural gas and coal, has not been studied. On the other hand, the high-order moments of tourism stocks are not fully described. However, several studies found exceptional returns for tourism stocks during crisis, such as extreme tail asymmetric risk [51] and speculative nature [52], are related to higher-order moments spillovers. Our study fills this gap by understanding high-order moments spillovers of oil, gas, coal, carbon, and tourism markets.

### 2.3 Carbon and tourism markets

Previous research has explored the connection between tourism and carbon emissions. Some studies have shown that tourism development reduces national carbon emissions [53,54]. For example, Brahmasrene and Lee [55] argued that tourism growth could result in lower carbon emissions, depending on the percentage of tourism in the GDP. Countries with more tourists and higher tourism density tend to have better environmental performance [56]. Additionally, some studies have concluded that while short-term tourism development may lead to increased emissions, it can ultimately contribute to long-term reductions in emissions driven by tourism [57]. This phenomenon is known as the Environmental Kuznets Curve (EKC) of tourism expansion [58]. Tourism development significantly negatively affects carbon emissions in some countries, such as Canada, the Czech Republic, and Turkey. In contrast, it positively and substantially impacts carbon emissions in other countries, including Italy, Luxembourg, and Slovakia [59]. These studies demonstrate a close relationship between carbon emissions and the tourism industry, but the conclusions are inconsistent, leading to uncertainty between the carbon market and tourism stocks. Currently, there is no literature addressing this aspect. Existing studies confirm that rising carbon prices increase energy consumption costs, which may lead to declining profits in related industries and exert pressure on stock prices [60]. Therefore, this paper aims to explain the changing characteristics of this relationship. These markets are important options for portfolio diversification [61–64].

## 3. Data and methodology

### 3.1 Data

In order to examine the relationship between the energy, carbon, and tourism markets, we selected the STOXX Global 1800 Travel & Leisure stock index to represent the global tourism stock market. This index consists of tourism companies from various European and Asian countries, most of which are US tourism companies. The EU carbon market is currently the largest and most established carbon market. For the carbon market, this study choses the settle price of the continuous futures contract of the European Union Allowances (EUA) in the European Climate Exchange (ECX) as the proxy for carbon emission allowance price. This paper selected the main components of the energy market structure: oil, gas, and coal, which are expressed in terms of WTI crude oil futures price, Natural Gas Futures Contract 1 (Dollars per Million Btu), and ICE Rotterdam Coal Futures price, respectively. These data were sourced from the Bloomberg database and cover daily observations from July 27, 2006, to November 10, 2022, with a total of 3,630 observations.

Then, based on the Glosten, Jagannathan, and Runkle GARCH (GJR) model, this paper estimates the high-order moments of energy, tourism, and carbon markets. We define a univariate GJRSK model. Here, The asset return *r*_*t*_ reflects the percentage change in the asset price at time *t*, providing insight into the profitability of holding the asset. Conditional volatility *h*_*t*_ indicates the level of risk associated with the asset by measuring the degree of variation in returns; higher values suggest greater uncertainty. Conditional skewness *s*_*t*_ reveals the asymmetry of the return distribution, where positive skewness indicates a prevalence of small losses with occasional large gains, while negative skewness suggests the opposite pattern. Lastly, conditional kurtosis *k*_*t*_ assesses the "tailedness" of the return distribution, with higher values indicating a greater likelihood of extreme outcomes. Together, these elements allow for a comprehensive understanding of market behavior, particularly by capturing higher-order moments that provide deeper insights into risks and potential returns beyond traditional mean-variance analysis. The formulation of the GJRSK model is given as follows.


$$r_{t}=\alpha_{0}+\alpha_{1}r_{t-1}+\varepsilon_{t}$$ (1)

$$h_{t}=\beta_{0}+\beta_{1}\varepsilon_{t-1}^{2}+\beta_{2}h_{t-1}+\beta_{3}\varepsilon_{t-1}^{2}I_{\left\{\eta_{t-1}<0\right\}}$$ (2)

$$s_{t}=\gamma_{0}+\gamma_{1}\eta_{t-1}^{3}+\gamma_{2}s_{t-1}+\gamma_{3}\eta_{t-1}^{3}I_{\left\{\eta_{t-1}<0\right\}}$$ (3)

$$k_{t}=\delta_{0}+\delta_{1}\eta_{t-1}^{4}+\delta_{2}k_{t-1}+\delta_{3}\eta_{t-1}^{4}I_{\left\{\eta_{t-1}<0\right\}}$$ (4)

$$\eta_{t}=h_{t}^{-\frac{1}{2}}\varepsilon_{t}$$ (5)

$$\eta_{t}|I_{t-1}∼g(0,1,s_{t},k_{t})$$ (6)

Here *α*_0_,*α*_1_ are parameters of the AR model and *β*_*i*_,*γ*_*i*_,*δ*_*i*_,*i* = 0, 1, 2, 3 are parameters of the GJRSK model. *I*_*A*_ is an indicator function that returns 1 if *A* is true and 0 otherwise. *g* is a probability density function with mean 0, variance 1, skewness *s*_*t*_, and kurtosis *k*_*t*_.

The probability density function *g*(0,1,*s*_*t*_,*k*_*t*_) of the GJRSK model can be given by Gram-Charlier expansion using Chebyshev-Hermite polynomials as follows:

$$g|\eta_{t}I_{t-1})=\frac{\phi (\eta_{t})\psi^{2}(\eta_{t})}{\Gamma_{t}}$$ (7)

$$\phi (\eta_{t})=\frac{1}{\sqrt{2\pi h_{t}}}\exp\left(\eta_{t}^{2}-h_{t}\right)$$ (8)

$$\psi (\eta_{t})=1+\frac{s_{t}}{3!}(\eta_{t}^{3}-3\eta_{t})+\frac{k_{t}-3}{4!}(\eta_{t}^{4}-6\eta_{t}^{2}+3)$$ (9)

$$\Gamma_{t}=1+\frac{s_{t}^{2}}{3!}+\frac{(k_{t}-3{)}^{2}}{4!}$$ (10)

The probability density function of *ε*_*t*_ is $f(\eta_{t}|I_{t-1})=h_{t}^{\frac{1}{2}}g(\eta_{t}|I_{t-1})$ because $\eta_{t}=h_{t}^{-\frac{1}{2}}\varepsilon_{t}$. Therefore, the log-likelihood function *l*_*t*_ without the constant term is obtained as:

$$l_{t}=-\frac{1}{2}ln\;h_{t}-\frac{1}{2}\eta_{t}^{2}+ln(\psi^{2}(\eta_{t}))-\Gamma_{t}$$ (11)

We can estimate each parameter of the GJRSK model by maximizing log-likelihood function *l*_*t*_.

Table 1 presents descriptive statistics of return, volatility, skewness, and kurtosis of three markets. The Phillips-Perron (PP) and Augmented Dickey-Fuller tests support the conclusion that all series are stationary at the 1% significance level. *** shows that the unit root test is significant at a 1% significance level. Therefore, these series can be used in the TVP-VAR-based analysis of spillovers.

**Table 1:** Descriptive statistics.

		Mean	SD	Min	Median	Max	ADF	PP
Returns	TL	0.000	0.011	-0.101	0.001	0.091	-52.261***	-52.105***
	EUA	0.001	0.031	-0.435	0.001	0.241	-61.028 ***	-61.058***
	Oil	-0.001	0.062	-3.060	0.001	0.320	-45.485***	-44.700 ***
	Gas	-0.000	0.034	-0.300	0.000	0.269	-64.752***	-64.839 ***
	Coal	0.000	0.024	-0.537	0.001	0.326	-57.967 ***	-58.087 ***
Volatility	TL	0.000	0.000	0.000	0.000	0.003	-7.067***	-8.605***
	EUA	0.001	0.001	0.000	0.001	0.027	-11.506***	-11.270 ***
	Oil	0.004	0.030	0.001	0.001	0.584	-9.817***	-10.164***
	Gas	0.001	0.001	0.000	0.001	0.013	-5.401***	-6.263***
	Coal	0.000	0.000	0.000	0.000	0.013	-18.025***	-17.983***
Skewness	TL	-0.109	0.052	-0.429	-0.119	0.429	-12.866***	-12.503***
	EUA	-0.043	0.015	-0.342	-0.041	0.101	-82.976***	-83.302***
	Oil	-7.924	592.426	-34938.680	0.110	7137.325	-74.105***	-74.119 ***
	Gas	0.051	0.047	-0.679	0.045	1.002	-27.244***	-27.287***
	Coal	-3.498	102.453	-5635.786	0.031	223.580	-40.103***	-40.086***
Kurtosis	TL	3.378	0.868	3.323	3.323	48.422	-60.347 ***	-60.348***
	EUA	3.503	0.001	3.503	3.503	3.553	-54.003 ***	-54.103***
	Oil	249.893	14775.21	4.261	4.262	890203.300	-60.241***	-60.241 ***
	Gas	3.438	0.686	3.303	3.328	27.251	-36.986***	-36.309 ***
	Coal	16.629	449.370	4.667	4.668	25694.430	-43.636***	-43.624***

Note

*** indicate significant at the 1% level.

### 3.2 Methodology

This paper utilizes the novel TVP-VAR frequency connectedness approach proposed by Chatziantoniou, Gabauer [23], which efficiently takes advantage of the essence of the previous work of Baruník and Křehlík [24] and Antonakakis, Chatziantoniou [25]. In this section, we first give a brief introduction of the TVP-VAR-based connectedness approach of Antonakakis, Chatziantoniou [25], which efficiently integrates the connectedness index of Diebold and Yilmaz [65] and the TVP-VAR model of Koop and Korobilis [66]. The TVP-VAR(p) can be presented as:

$$y_{t}=\Phi_{1t}y_{t-1}+\Phi_{2t}y_{t-2}+\cdots +\Phi_{pt}y_{t-p}+\epsilon_{t}\;\epsilon_{t}∼N(0,\Sigma_{t})$$ (12)

where *y*_*t*_ and *ϵ*_*t*_ are *N*×1 vectors, Σ_*t*_ the *N*×*N* time-varying variance-covariance matrix and Φ_*it*_,*i* = 1,…, *p* represents the *N*×*N* time-varying VAR coefficient. With the matrix lag-polynomial $\Phi (L)=[I_{N}-\Phi_{1t}L-\cdots -\Phi_{pt}L^{p}]$ and the Wold representation theorem, the stationary TVP-VAR process can be rewritten as a TVP-VMA(∞): *x*_*t*_ = Ψ(*L*)*ϵ*_*t*_ where Φ(*L*) = [Ψ(*L*)]^−1^. As *Ψ*(*L*) includes infinite lags, it is approximated by computed Ψ_*h*_ at *h* = 1,…,*H* horizons [23].

With the TVP-VMA coefficients Ψ_*h*_, we can compute the generalized forecast error variance decomposition (GFEVD) which can be interpreted as the effect that a shock in variable *j* has on variable *i* in terms of its forecast error variance and can be written as:

$$C_{ijt}(H)=\frac{(\Sigma_{t}{)}_{jj}^{-1}\sum_{h=0}^{H}((\Psi_{h}\Sigma_{t}{)}_{ijt}{)}^{2}}{\sum_{h=0}^{H}(\Psi_{h}\Sigma_{t}\Psi_{h}^{\prime }{)}_{ii}}$$ (13)

$${\tilde{C}}_{ijt}(H)=\frac{C_{ijt}(H)}{\sum_{k=1}^{N}C_{ijt}(H)}$$ (14)

where ${\tilde{C}}_{ijt}(H)$ represents the contribution of the *j*th variable to the variance of the forecast error of the *i*th variable at horizon *H*. With row normalization of ${\tilde{C}}_{ijt}(H)$ we have $\sum_{i=1}^{N}{\tilde{C}}_{ijt}(H)=1$ and $\sum_{j=1}^{N}\sum_{i=1}^{N}{\tilde{C}}_{ijt}(H)=N$.

Thus, we are able to compute all the connectedness measures including:

Net pairwise directional connectedness:

$$NPDC_{ijt}(H)={\tilde{C}}_{ijt}(H)-{\tilde{C}}_{jit}(H)$$ (15)

It means that variable *j* influences variable *i* more (less) than vice versa with *NPDC*_*ijt*_(*H*)>(<)0.

Total directional connectedness TO others:

$$TO_{it}(H)=\sum_{i=1,i\neq j}^{N}{\tilde{C}}_{jit}(H)$$ (16)

It measures how much of a shock in variable *i* is transmitted to all other variables *j*.

Total directional connectedness FROM others:

$$FROM_{it}(H)=\sum_{j=1,i\neq j}^{N}{\tilde{C}}_{ijt}(H)$$ (17)

It measures how much variable *i* is receiving from shocks in all other variables *j*.

Net total directional connectedness:

$$NET_{it}(H)=TO_{it}(H)-FROM_{it}(H)$$ (18)

It represents the difference between the total directional connectedness *TO* others *TO*_*it*_(*H*) and the total directional connectedness *FROM* others *FROM*_*it*_(*H*), which can be interpreted as the net influence variable *i* has on the corresponding volatility transmission network. It is regarded as a net transmitter (receiver) of shocks with *NET*_*it*_(*H*)>(<)0 indicating that variable *i* influences all others *j* more (less) than being influenced by them.

Total averaged connectedness index:

$$TCI_{t}(H)=N^{-1}\sum_{i=1}^{N}TO_{it}(H)=N^{-1}\sum_{i=1}^{N}FROM_{it}(H)$$ (19)

It depicts the average impact a shock in one variable has on all others, thus measuring the degree of network interconnectedness and market risk [23].

Unifying the TVP-VAR connectedness framework with the spectral representation of variance decompositions introduced by the BK model [67], we can explore the volatility connectedness between variables of interest in the frequency domain. With the frequency response function, $\Psi (e^{-i\omega })=\sum_{h=0}^{\infty }e^{-i\omega h}\Psi_{h},i=\sqrt{-1}$ where and *ω* represents the frequency to continue with the spectral density of *y*_*t*_ at frequency *ω*. The spectral density of *y*_*t*_ over *ω* can be defined as a Fourier transformation of the TVP-VMA(∞):

$$S_{y}(\omega )=\sum_{h=-\infty }^{\infty }E(y_{t}y_{t-h}^{\prime })e^{-i\omega h}=\Psi (e^{-i\omega h})\Sigma_{t}\Psi^{\prime }(e^{+i\omega h})$$ (20)

The frequency GFEVD, as the combination of the spectral density and the GFEVD, therefore, can be computed as:

$$C_{ijk}(\omega )=\frac{{\left(\Sigma_{t}\right)}_{jj}^{-1}{|\sum_{h=0}^{\infty }{\left(\Psi (e^{-i\omega h})\Sigma_{t}\right)}_{ijt}|}^{2}}{\sum_{h=0}^{\infty }{\left(\Psi (e^{-i\omega h})\Sigma_{t}\Psi (e^{i\omega h})\right)}_{ii}}$$ (21)

$${\tilde{C}}_{ijk}(\omega )=\frac{C_{ijt}(\omega )}{\sum_{k=1}^{N}C_{ijt}(\omega )}$$ (22)

We further aggregate all frequencies within a range of interest, ${\tilde{\theta }}_{ijt}(d)=\int_{a}^{b}{\tilde{\theta }}_{ijt}(\omega )d\omega$, where *d* = (*a, b*): *a, b* ∈ (−*π, π*), *a* < *b*, and then we can calculate all the frequency connectedness measures that provide information about spillovers in a certain frequency range *d*:

$$NPDC_{ijt}(d)={\tilde{C}}_{ijt}(d)-{\tilde{C}}_{jit}(d)$$ (23)

$$TO_{it}(d)=\sum_{i=1,i\neq j}^{N}{\tilde{C}}_{jit}(d)$$ (24)

$$FROM_{it}(d)=\sum_{j=1,i\neq j}^{N}{\tilde{C}}_{ijt}(d)$$ (25)

$$NET_{it}(d)=TO_{it}(d)-FROM_{it}(d)$$ (26)

Here, FROM represents the total spillover effects received by a market from other markets, reflecting the extent to which external market shocks influence the market. In contrast, TO indicates the total spillover effects transmitted by a market to other markets, demonstrating the market’s influence and significance within the overall market system. Finally, NET represents the net spillover of that market, revealing its role in the entire network and distinguishing whether it is a net receiver or a net transmitter. These indicators help to comprehensively assess the interactions between markets and potential risks, thereby providing important insights for decision-making.

## 4. Empirical results

### 4.1 Static spillovers

This section first investigates the static connectedness among energy, carbon, and tourism markets in the time domain. To get an overall view of the connectedness effects, we report the estimation results by presenting the connectedness of return, volatility, skewness, and kurtosis in Table 2.

**Table 2:** Full-sample connectedness in time- and frequency- domain.

		TL	EUA	Oil	Gas	Coal	FROM
Return	TL	85.94 (59.91) [26.03]	4.21 (2.88) [1.33]	6.71 (4.45) [2.25]	1.44 (0.96) [0.48]	1.70 (1.38) [0.32]	14.06 (9.68) [4.38]
	EUA	3.94 (2.70) [1.23]	87.70 (67.48) [20.22]	4.16 (3.25) [0.91]	1.22 (0.79) [0.43]	2.99 (2.56) [0.43]	12.30 (9.31) [3.00]
	Oil	5.64 (4.11) [1.53]	3.62 (2.73) [0.89]	86.37 (66.65) [19.72]	2.36 (1.82) [0.54]	2.01 (1.52) [0.49]	13.63 (10.18) [3.46]
	Gas	1.70 (1.18) [0.52]	1.19 (0.87) [0.32]	2.62 (2.11) [0.51]	93.11 (73.19) [19.92]	1.38 (1.10) [0.28]	6.89 (5.26) [1.63]
	Coal	1.63 (0.96) [0.66]	3.04 (2.15) [0.89]	2.74 (1.90) [0.84]	1.34 (0.93) [0.41]	91.25 (66.52) [24.73]	8.75 (5.94) [2.81]
	TO	12.90 (8.96) [3.95]	12.06 (8.63) [3.43]	16.23 (11.71) [4.52]	6.36 (4.50) [1.85]	8.09 (6.56) [1.52]	55.64 (40.36) [15.27]
	NET	-1.16 (-0.72) [-0.44]	-0.25 (-0.68) [0.43]	2.60 (1.53) [1.06]	-0.53 (-0.76) [0.23]	-0.66 (0.63) [-1.28]	
Volatility	TL	82.25 (4.44) [77.81]	2.97 (0.15) [2.82]	10.57 (0.54) [10.02]	1.05 (0.08) [0.97]	3.17 (0.11) [3.06]	17.75 (0.88) [16.87]
	EUA	3.01 (0.08) [2.93]	81.42 (2.95) [78.47]	7.76 (0.53) [7.23]	3.35 (0.12) [3.23]	4.47 (0.16) [4.31]	18.58 (0.89) [17.70]
	Oil	4.17 (0.24) [3.93]	2.59 (0.18) [2.41]	83.86 (0.68) [83.18]	4.52 (0.37) [4.15]	4.85 (0.22) [4.63]	16.14 (1.01) [15.13]
	Gas	1.27 (0.07) [1.20]	3.60 (0.09) [3.50]	4.53 (0.24) [4.29]	85.11 (1.08) [84.03]	5.50 (0.15) [5.36]	14.89 (0.55) [14.34]
	Coal	2.11 (0.17) [1.94]	2.23 (0.25) [1.98]	6.16 (0.34) [5.82]	3.07 (0.28) [2.79]	86.43 (12.38) [74.05]	13.57 (1.04) [12.53]
	TO	10.56 (0.56) [10.00]	11.38 (0.67) [10.71]	29.02 (1.66) [27.36]	11.99 (0.85) [11.14]	17.99 (0.63) [17.36]	80.93 (4.37) [76.56]
	NET	-7.19 (-0.32) [-6.87]	-7.20 (-0.21) [-6.99]	12.88 (0.65) [12.23]	-2.90 (0.30) [-3.20]	4.42 (-0.41) [4.83]	
Skewness	TL	63.63 (26.88) [36.75]	6.52 (1.58) [4.94]	10.37 (1.98) [8.39]	11.96 (6.33) [5.63]	7.52 (1.84) [5.68]	36.37 (11.73) [24.64]
	EUA	5.47 (2.39) [3.08]	82.90 (63.66) [19.24]	3.65 (1.73) [1.92]	4.98 (1.94) [3.04]	3.01 (0.57) [2.44]	17.10 (6.63) [10.47]
	Oil	6.29 (1.89) [4.41]	1.46 (0.55) [0.91]	63.71 (35.38) [28.33]	5.24 (1.55) [3.69]	23.30 (5.00) [18.30]	36.29 (8.97) [27.31]
	Gas	13.69 (8.17) [5.52]	2.68 (1.25) [1.42]	6.87 (3.07) [3.80]	71.74 (50.32) [21.41]	5.02 (1.01) [4.01]	28.26 (13.51) [14.75]
	Coal	5.39 (0.55) [4.84]	1.21 (0.15) [1.06]	21.70 (2.78) [18.93]	4.73 (0.44) [4.29]	66.97 (23.68) [43.28]	33.03 (3.91) [29.12]
	TO	30.85 (13.00) [17.84]	11.86 (3.53) [8.34]	42.60 (9.56) [33.04]	26.91 (10.26) [16.64]	38.85 (8.42) [30.43]	151.06 (44.77) [106.29]
	NET	-5.52 (1.27) [-6.79]	-5.24 (-3.11) [-2.13]	6.31 (0.58) [5.72]	-1.36 (-3.25) [1.89]	5.81 (1.27) [1.31]	
Kurtosis	TL	61.49 (41.10) [20.39]	14.10 (5.39) [8.71]	7.99 (3.34) [4.65]	11.69 (2.84) [8.85]	4.73 (2.15) [2.58]	38.51 (13.72) [24.79]
	EUA	10.67 (2.76) [7.91]	40.74 (26.96) [13.78]	21.65 (14.37) [7.28]	12.93 (2.97) [9.96]	14.01 (10.84) [3.17]	59.26 (30.93) [28.32]
	Oil	1.59 (0.07) [1.51]	15.45 (5.20) [10.25]	58.05 (30.04) [28.01]	4.07 (0.14) [3.93]	20.84 (9.68) [11.16]	41.95 (15.09) [26.86]
	Gas	8.47 (2.44) [6.03]	10.30 (2.96) [7.34]	10.05 (2.35) [7.70]	66.36 (36.39) [29.96]	4.82 (1.68) [3.14]	33.64 (9.44) [24.20]
	Coal	1.54 (0.29) [1.25]	15.18 (3.96) [11.23]	26.82 (7.39) [19.43]	3.96 (0.25) [3.70]	52.50 (25.54) [26.96]	47.50 (11.89) [35.61]
	TO	22.26 (5.57) [16.69]	55.03 (17.52) [37.52]	66.51 (27.45) [39.07]	32.65 (6.19) [26.45]	44.40 (24.35) [20.05]	220.86 (81.08) [139.78]
	NET	-16.25 (-8.15) [-8.10]	-4.22 (-13.42) [9.19]	24.57 (12.36) [12.21]	-1.00 (-3.25) [2.25]	-3.10 (12.46) [-15.55]	

Note: The data in () and [] represent connectedness measures in the short and long-term, respectively. The time horizon of the short-term measure is less than 5 days, and that of the long-term measure is more than 5 days.

Firstly, the total connectedness of return, volatility, skewness, and kurtosis among the three markets is measured at 55.64, 80.93, 151.06, and 220.86, respectively, indicating a strong dependence among the three markets. We can find that the spillover effect of oil on the return and volatility of tourism stocks is robust, with 6.71 and 10.57, respectively. It suggests that energy markets and tourism stock returns interact closely, similar to Qin, Chen [68]’s findings. The strong link between oil and tourism stock is also easy to understand and could be due to fundamentals: the close link between crude oil and travel consumption. Crude oil is the main fuel power for passenger transport, and rising oil prices lead to higher operating costs and reduced corporate cash flow [69]. To a certain extent, other leisure tourism industries and passenger transport services are complementary. Rising travel costs may reduce people’s willingness to travel, eventually leading to a decline in tourism and leisure return and stock prices [70,71].

Secondly, the total spillover of volatility is the largest; the return is slightly smaller; the total spillover index of higher-order moments (skewness and kurtosis) is relatively small relative to volatility. In contrast to the strong correlation between the first- and second-moments (return and volatility), the spillovers among energy, carbon, and tourism markets remain when the low probability event occurs. However, different markets react differently. Oil is the most significant net contributor to other markets, with the net spillover of 2.6, 12.8, 6.31, and 24.57 for return, volatility, skewness, and kurtosis, respectively. Tourism stocks are net recipients of return spillovers, while the volatility shows that the carbon market is the net recipient. In the spillover effects of skewness and kurtosis, the carbon market is always the net recipient. That is to say, even in a low-probability event, the carbon market will still be subject to energy and tourism stock spillover.

As mentioned above, markets react differently to exogenous shocks at heterogeneous frequencies. Investors would have different expectations of investment returns from heterogeneous investment horizons. We report the connectedness between them at different time frequencies in Table 2. The short-term return connectedness is 40.36, while the long-term return connectedness decreases to 15.27. The short-term volatility connectedness is only 4.37, and the long-term volatility connectedness increases to 76.56. The return spillovers diminish over time, while the volatility is contrary, which means that the short-term return is the main part of spillovers, while the long-term volatility determines the spillover effect. Skewness and kurtosis both show an increasing trend with the development from short-to long-term. In other words, the higher-order moments spillovers become stronger in the long run. Regardless of the frequency band, oil is a net emitter of spillover effects, and carbon market return and volatility are net recipients. Skewness and kurtosis convey much information. The carbon market has gradually changed from a short-term kurtosis spillover effect receiver (-13.42) to a long-term transmitter (9.19), while the tourism stocks have gradually changed from a short-term skewness spillover effect transmitter (1.27) to a long-term receiver (-6.79). In this regard, we call on investors to pay attention to the carbon market in the short term (within 5 days) under extreme scenarios and in the long term (more than 5 days) to carefully consider portfolio strategies that have been incorporated into the tourism stocks.

### 4.2 Net spillover network

**Fig 1:**
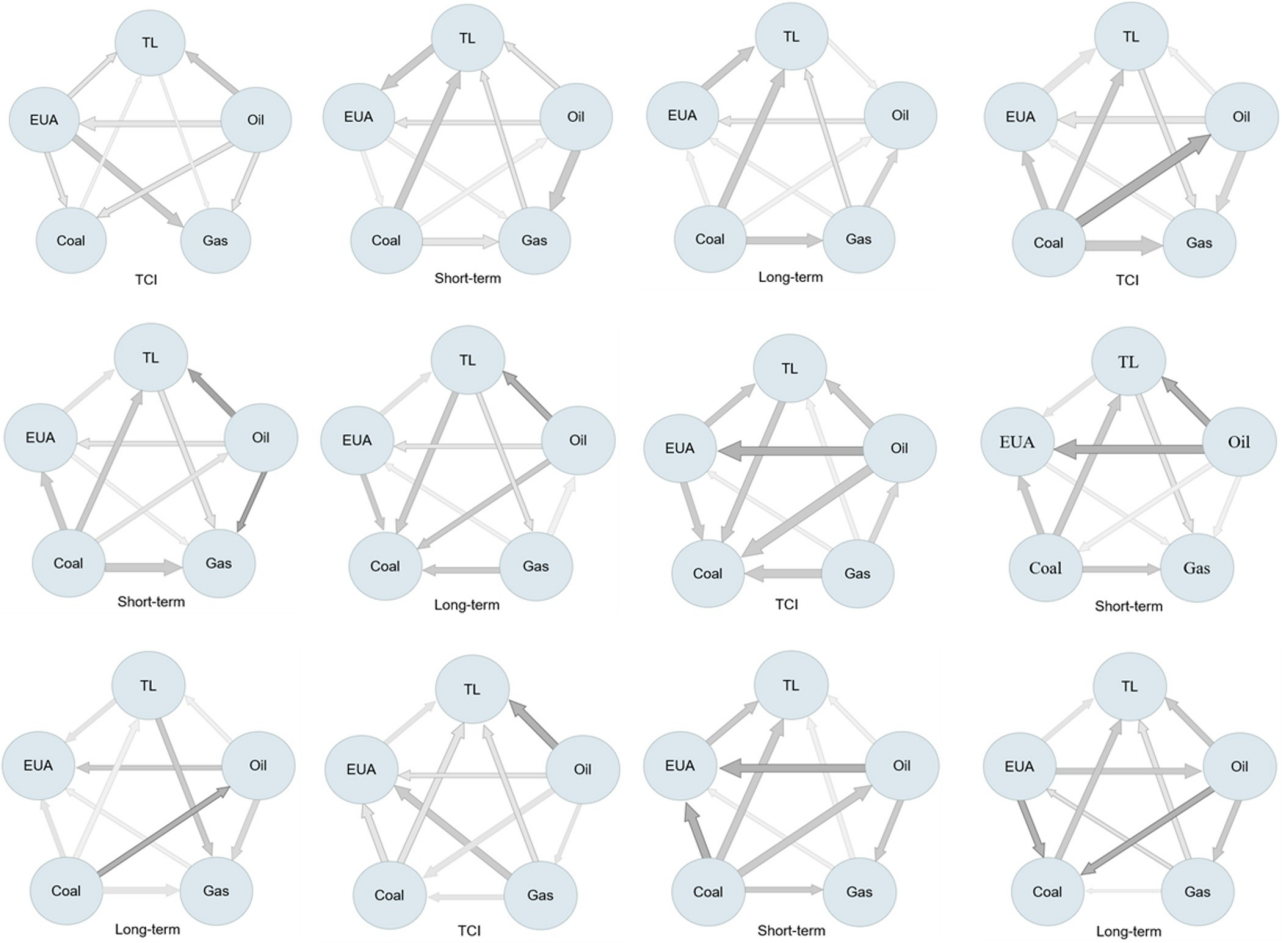
The net spillover network of energy, carbon, and tourism markets. (a) return (b) volatility (c)skewness (d) kurtosis.

Fig 1 shows the net spillover network of return, volatility, skewness, and kurtosis. It can be seen from Fig 1A and 1B that the energy market is a net spillover exporter in both return and volatility, and the tourism stock is a net spillover recipient. After decomposing the net directional connectedness to short- and long-term, it can be observed that oil returns accept the spillover effect of the tourism stock in the long-term. Unlike the above, the skewness net spillover direction shows that in the long-term, the carbon market receives the asymmetric spillover effects of the other two markets. On the one hand, energy combustion is the main source of carbon emissions. Reducing energy prices will increase energy consumption, which usually leads to higher carbon emissions demand and costs [29]. On the other hand, although carbon emissions caused by short-term tourism activities impact carbon prices, in the long term, it will limit tourism. For example, in the carbon market, due to price restrictions, tourism companies spend more to obtain carbon emission permits, which is not conducive to earnings and puts pressure on company stock prices [72].

By observing the kurtosis spillover effect in Fig 1D, energy, including oil, natural gas, and coal, are all net spillover exporters. At the same time, the carbon market acts as an intermediary transmission, and the tourism stocks are the net recipient. Even in the long-term, tourism stocks are still the recipients, which means that tourism stocks bear more spillover risks at extreme times. According to Diebold and Yilmaz [73], after huge crisis events, shocks can quickly spread to assets and markets under high volatility conditions. There is an evident risk contagion in the global stock industry. The risk of other markets is easily spread to related industries, which can explain the connectedness of different markets at the leptokurtosis and fat-tail moment. Therefore, in the extreme upward (downward) period, the tourism stock market needs to be more guarded against energy and carbon market risks.

### 4.3 Dynamic spillover effect

**Fig 2:**
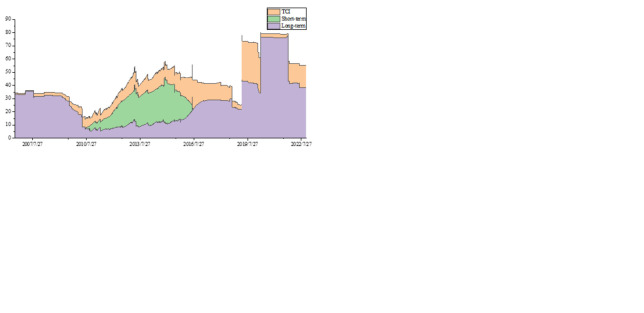
Total connectedness of return, volatility, skewness, and kurtosis. (a) return (b) volatility (c) skewness (d) kurtosis.

Fig 2 shows the dynamic evolution of the short- and long-term spillover effects. The results show that the return spillovers have apparent time-varying connectedness, which is more robust in the short-term. Volatility spillovers almost do not have a short-term effect but are more significant in the long-term. During major events, the volatility spillover effect increases significantly. After the COVID-19 pandemic, the skewness and kurtosis spillover effects increased to 80%, rising to a very high level. As a major crisis, the epidemic significantly exacerbated the higher-order moments’ spillovers of energy, carbon, and tourism markets. The kurtosis spillover effect maintains a higher level in the long run, showing the leptokurtosis and fat-tail characteristics, which indicates the vulnerability of long-term risk resistance. The reasonableness of our results depends on the fact that real-time reports caused by the epidemic have exacerbated the fear and panic of corporations and investors. Temporarily closing the company by reducing activities and production capacity will ultimately harm the company’s profitability. Therefore, investors become risk-averse in investment decisions, accompanied by higher-order moments spillovers in three markets. After the outbreak of the Russia-Ukraine conflict in February 2022, it can be found that the skewness and kurtosis spillovers remain at a high level of 50%. Compared with major crises such as the Sino-US trade war and the epidemic, the Russia-Ukraine conflict has more significantly exacerbated the spillovers in high-order moments. Therefore, this continuous geopolitical event has significant impacts not only on returns and volatility spillover effects but also on the higher-order moments.

**Fig 3:**
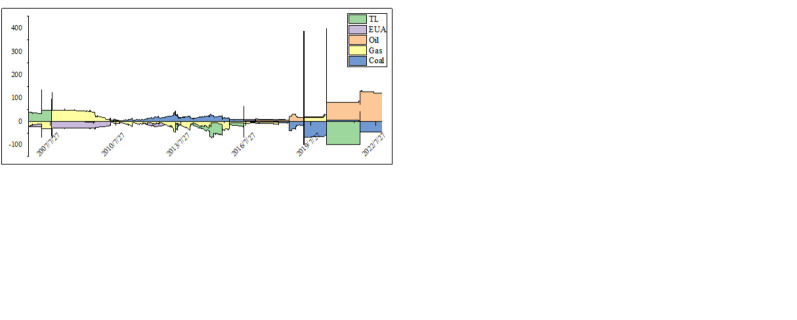
Net connectedness of return, volatility, skewness, and kurtosis. (a) return (b) volatility (c) skewness (d) kurtosis.

Fig 3 shows the dynamic evolution of the net spillover effects. It can be seen in Fig 3A that the return spillover effects of oil and tourism stocks on other markets have increased significantly after the outbreak of the COVID-19 epidemic. Among them, the spillover effects of oil have reached their peak within ten years, rapidly weakened after the epidemic outbreak, and are mainly manifested as long-term volatility spillover effects. It once again proves that during the COVID-19 pandemic, the restrictive measures taken by government-authorized non-profit organizations have had a significant impact on economic development, such as the collapse of oil prices and the four-time triggering of the U.S. stock market, resulting in investors suffering heavy losses in a brief period [74]. As for volatility, the spillover effect of oil was powerful before the financial crisis. At the same time, the carbon market accepted much spillover effects from other markets. It shows that the early mode of promoting development, known as the energy-consumption mode, led the carbon market to rely heavily on energy. Obviously, this tendency has slowed down in the next ten years. In the last three years, the net spillover effect of volatility has two peaks during the COVID-19 pandemic and the Russo-Ukrainian war, which requires skewness and kurtosis spillovers to provide more information.

By observing the net spillover effect of skewness in Fig 3C, we find that the net spillover effect of the energy market peaked at the beginning of the COVID-19 epidemic. Specifically, the coal market has an asymmetric spillover effect on other markets. The carbon market is the recipient of skewness spillovers. Even in the mid-period of the epidemic, we observe that the carbon market still accepts skewness spillovers from the coal market. Increasingly complex competitive relationships have tightened restrictions on interactions between coal importers. Therefore, a country’s conflict, sanctions, or coal import policies during the crisis period will affect competitors’ competition and the coal trade’s stability [75]. Coal-related carbon dioxide emissions are still the largest source of fuel-related carbon emissions, leading to considerable fluctuations in carbon prices.

According to the kurtosis spillover effect in Fig 3D, there were two kurtosis net oil spillover peaks during the COVID-19 epidemic and the Russo-Ukrainian war, and the former was stronger. After the pandemic, it is mainly tourism stocks that accept kurtosis spillovers from other assets, which indicates that the pandemic has affected the relationship between oil and the stock market. Mensi, Vo [76] found that the spillover effect between crude oil and the stock market increased during the spread of the COVID-19 pandemic. They also indicated that the beneficial effect of diversified oil assets declined during this period. Given the increased extreme risk contagion of oil and tourism stocks, oil has not provided more protection for stock declines. After the Russo-Ukrainian War, oil was still the sender of the spillover effect. However, the recipient of the kurtosis spillover effect switched to coal, which means that the Russo-Ukrainian War led to extreme risk transmission within the energy market. The Russia-Ukraine war has exacerbated the risk of disruption to the global energy supply chain. It not only causes sharp fluctuations in energy prices in a short period, leading to geopolitical tensions, but also disrupts global energy supply and economic and trade order. In the long run, it will affect the global political and economic landscape [77].

**Fig 4:**
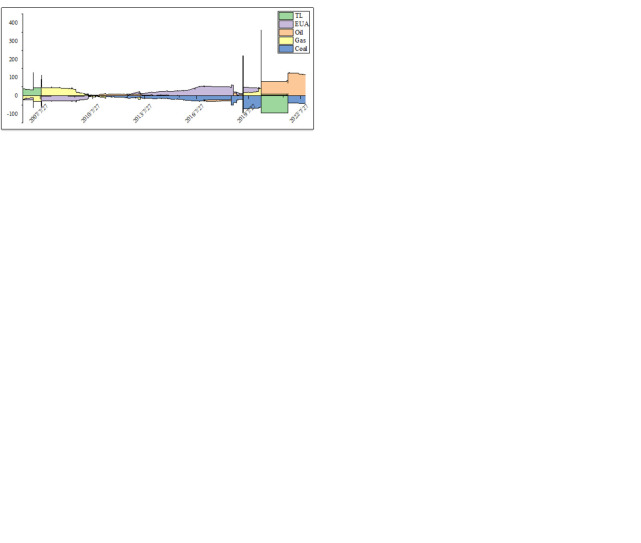
Net connectedness of return, volatility, skewness, and kurtosis in short- and long-term frequencies. (a) return in short-term frequencies (b) return in long-term frequencies (c) volatility in short-term frequencies (d) volatility in long-term frequencies (e) skewness in short-term frequencies (f) skewness in long-term frequencies (g) kurtosis in short-term frequencies (h) kurtosis in long-term frequencies.

Fig 4 shows the short-term and long-term dynamic evolution of the net spillover effects. According to Fig 4A and 4D, the short-term net spillover of return is stronger, while the long-term effect of volatility spillover is more obvious. In the short-term, after the COVID-19 epidemic, the oil spillover is mainly return-dominated. In the long term, the sender of the spillover is natural gas, and the receiver is the alternative emergence of various assets. The pandemic has led to frequent spillovers among the return of energy, carbon market, and tourism stocks. Fig 4E–4H show the net spillover effects of skewness and kurtosis in different frequency domains. The skewness net spillover effect reflects the asymmetric risk contagion dominated by the energy market (coal and natural gas) in the short-term and the tourism stock market in the long term after the COVID-19 epidemic. During this period, the carbon market is the net recipient of skewness spillovers. It proves that skewness spillover effects exist in different frequency domains after a major crisis. Jiatong Liu et al. (2023) also said that most industries’ dependence structure between the carbon trading market and industry stocks is asymmetric. There are many mutation structures with significant risks in extreme cases. During the Russo-Ukrainian War, the long-term kurtosis spillover of oil lasted for a long time, and the transmission to other assets showed a moderate upward trend in the later period.

### 4.4 Robustness checks

In this section, we further replaced the GJRSK model with the GARCHSK model, replaced STOXX Global 1800 Travel & Leisure with FTSE Global Travel & Leisure, WTI crude oil was replaced with Brent crude oil, and Rotterdam Coal was replaced with Newcastle Coal Futures to reestimate the main empirical results and compare them with previous analyses. Table 3 shows the empirical results based on the new data and model. In general, the empirical results in Table 3 are consistent with those in Table 2. The results show that these estimates are similar, confirming the robustness and validity of our results under the new model and data.

**Table 3:** Full-sample connectedness based on TVP-DY model.

		TL	EUA	Oil	Gas	Coal	FROM
Return	TL	86.8	3.9	6.3	1.4	1.5	13.2
	EUA	3.6	88.5	4.8	1.1	2.1	11.5
	Oil	6.2	4.3	84.7	1.9	2.9	15.3
	Gas	1.2	1.1	2.2	94.4	1.1	5.6
	Coal	1.7	1.9	3.4	1.4	91.6	8.4
	TO	12.6	11.3	16.6	5.8	7.6	53.9
	NET	-0.5	-0.2	1.3	0.2	-0.7	
Volatility	TL	84.2	3.0	10.4	1.1	1.3	15.8
	EUA	5.4	90.2	2.1	1.6	0.8	9.8
	Oil	9.5	3.6	82.0	2.6	2.3	18.0
	Gas	1.1	1.1	1.9	94.5	1.3	5.5
	Coal	1.7	1.3	2.6	3.3	91.2	8.8
	TO	17.7	9.1	17.0	8.5	5.8	58.0
	NET	1.9	-0.8	-1.0	3.0	-3.1	
Skewness	TL	88.1	2.5	6.3	1.6	1.5	11.9
	EUA	2.3	93.5	1.0	0.6	2.7	6.5
	Oil	2.7	1.4	93.9	0.9	1.1	6.1
	Gas	2.2	1.3	1.7	93.7	1.1	6.3
	Coal	0.2	0.1	0.4	0.6	98.8	1.2
	TO	7.4	5.3	9.4	3.6	6.3	32.0
	NET	-4.5	-1.2	3.3	-2.7	5.1	
Kurtosis	TL	85.1	2.2	7.2	1.4	4.1	14.9
	EUA	2.0	92.4	3.5	0.7	1.4	7.6
	Oil	2.4	2.2	90.2	0.5	4.7	9.8
	Gas	1.7	1.3	4.1	90.1	2.7	9.9
	Coal	1.3	1.7	5.6	1.3	90.1	9.9
	TO	7.3	7.4	20.3	4.0	12.9	52.0
	NET	-7.5	-0.1	10.4	-5.8	3.0	

## 5. Conclusions and implications

Based on the GJRSK model, we estimate the high-order moments of energy, carbon, and tourism markets. Then, we use a novel TVP-VAR time-frequency connectedness approach proposed by Chatziantoniou, Gabauer [23] to reveal the time-frequency connectedness of three markets. The empirical results show that:

Firstly, the static spillover results show a strong connectedness among the energy, carbon, and tourism markets and robust oil return and volatility spillovers to tourism stocks. Focusing on the higher-order moments’ spillovers, we found that the carbon market accepted stronger skewness and kurtosis spillovers. After decomposing the total spillover index into short- and long-term, the results show that return and volatility spillovers are more significant in the short term, while skewness and kurtosis spillovers have greater long-term effects.

Secondly, the net spillover connectedness network shows that the energy market is the net exporter of return and volatility spillovers, and the tourism stock is the net recipient. For the skewness spillovers, the carbon market is affected by the asymmetric effects of the other two markets. The kurtosis spillover effect mainly spills from the energy market to the tourism stock market, indicating that the risk contagion between energy and financial markets at extreme times, and even in the long-term tourism stock also bears more spillover risks.

Thirdly, observing the dynamic spillover effect, it is found that the three markets have time-varying connectedness, which is significantly enhanced during the crisis, and the net spillover position has also changed. Specifically, the short-term skewness spillover of the COVID-19 pandemic is dominated by the energy market, while tourism stocks dominate the long-term. During the Russo-Ukrainian War period, oil was the sender of the kurtosis spillover effect, while the recipient changed to coal, which means that the Russo-Ukrainian War led to extreme risk transmission within the energy market. This ongoing geopolitical event has a significant impact on returns and volatility spillovers and the connectedness of high-order moments spillovers. This finding is novel, and no other paper confirmed the impact of the Russo-Ukrainian conflict on the high-order moments spillovers of energy and financial markets.

The findings of this study have important implications for policymakers, investors, and industry practitioners in energy, tourism, and carbon markets. Firstly, the research reveals the spillover effects among the tourism, energy, and carbon markets for the first time, so policymakers should note the connections between them. Fluctuations in energy prices directly affect the operational costs of tourism businesses and indirectly impact the sustainability of the tourism industry through changes in the carbon market. Therefore, when formulating policies, policymakers should pay particular attention to the sustainability of the tourism market and ensure that it complements energy policies to enhance overall market stability. Secondly, investors should closely monitor the vulnerability of the carbon and tourism markets under extreme shocks, especially during periods of heightened market volatility. Fluctuations in the energy market may lead to rising carbon prices, increasing the operational costs for tourism companies and affecting their profitability. Investors need to dynamically adjust their portfolios, focusing on the interactions between the energy, tourism, and carbon markets to achieve higher returns. Additionally, considering the different responses of the tourism and carbon markets under varying economic conditions, investors should design hedging strategies to mitigate risks between the tourism sector and the carbon market. Thirdly, industry practitioners should flexibly adjust their operational strategies based on the interactions among the energy, tourism, and carbon markets to adapt to the impacts of energy changes on the carbon market. Changes in energy costs can significantly affect the pricing and demand for tourism products, influencing the performance of the tourism market. Therefore, when formulating business strategies, industry practitioners should comprehensively consider the interrelationships among the three markets to seize opportunities and enhance their competitiveness and resilience in the tourism and carbon markets. By implementing these comprehensive strategies, stakeholders can better navigate uncertain market environments and achieve sustainable development goals.

However, this study still has some limitations. Due to data availability and pricing transparency constraints, it relied on a single overall index of the tourism market and could not cover more specific submarkets, which may have prevented us from fully elucidating the diversity of roles within the tourism subsector. Future research could address this limitation by adopting an index compilation approach, collecting and analyzing data from related industries, and constructing tourism submarket data indicators using a market capitalization weighting method. This would provide a more comprehensive understanding of the diversity within the entire tourism industry.
